# Digital twin analysis of graphene–silicon solar Schottky-heterojunction cell efficiency for terrestrial photovoltaics

**DOI:** 10.1038/s41598-026-48915-3

**Published:** 2026-04-20

**Authors:** Praveen Kumar Kanti, H. G. Prashantha Kumar, H. I. Elsaeedy, V. Vicki Wanatasanappan, Gabr Goshu Syum, Mohamed Bechir Ben Hamida

**Affiliations:** 1 Institute of Power Engineering, Universiti Tenaga Nasional, IKRAM-UNITEN, Jalan, 43000 Selangor Malaysia; 2 Centre for Research Impact & Outcome, Chikara University Institute of Engineering and Technology, Chitkara University, Rajpura 140401, Punjab, India; 3 Digital Twin lab, Department of Aerospace Engineering, Dayananda Sagar University (DSU), Bangalore, 560056 India; 4 Department of Physics, College of Science, King Khalid University, Abha, 61413 Saudi Arabia; 5 Faculty of Mechanical and Industrial Engineering, EiT-M, Mekelle University, P. O. Box 231, Mekelle, 7000 Tigray Ethiopia; 6 Centre of Excellence in Studies in Oil and Gas Engineering and Technology(CS-OGET), Eduardo Mondlane University, Bairro Luís Cabral, MoçambiqueMaputo, Mozambique; 7 Deanship of Scientific Research, Imam Mohammad Ibn Saud Islamic University (IMSIU), Riyadh, Saudi Arabia

**Keywords:** Graphene–Silicon Schottky heterojunction, Radiation Tolerance, Digital Twin, Terrestrial Photovoltaics, Energy science and technology, Materials science, Optics and photonics, Physics

## Abstract

Graphene–silicon (Gr–Si) Schottky heterojunction solar cells provide a promising solution for radiation-tolerant and efficient photovoltaic devices. The present study provides the combined predictive modelling and physical characterization to evaluate the degradation assessment of Gr–Si solar cells. The results show Gr–Si devices successfully retain over 84% of their original power conversion efficiency (PCE) at a radiation intensity in terms of fluence in the order of 10^15^ particles cm^−2^, compared to only 55% for silicon cells alone. X-ray diffraction (XRD), Raman spectroscopy, and Atom Probe Tomography (APT) showed that graphene retains the interface integrity and is efficient in suppressing radiation-induced imperfections. A physics-informed semi-empirical digital twin (DT) assessment carried out using synthetic degradation datasets and Random Forest Regression, performing prediction efficiency above 96% (R^2^ > 0.96). Degradation mathematical models were also formulated to describe the characteristics of open-circuit voltage (V_oc_), short-circuit current density ​(J_sc_)​, Fill Factor (FF), and Power Conversion Efficiency (PCE) for defined duration. The experimental validation and machine learning combined assessment provides a robust methodology for real-time monitoring and life-cycle prediction of photovoltaic devices in rich-radiation environments.

## Introduction

The importance of solar energy as a long-duration power source with stable and scalable power over long periods of time has increased in terrestrial photovoltaic missions^1^. Solar photovoltaic (PV) systems that are based on crystalline silicon (c-Si) prevail in terrestrial applications of photovoltaic systems since the technology is mature, has established manufacturing methods, and is cost-effective^2,3^. But, on earth, the photovoltaic conditions are typified by continuous exposure to high-energy radiation caused by events of solar particles, galactic cosmic rays, and trapped charged particles, which severely deteriorate the performance of the devices with time^4,5^. Displacement damages caused by radiation cause the creation of point defects and defect clusters in the silicon lattice that serve as non-radiative recombination centres, which decrease the minority carrier lifetime and diffusion length, and consequently the photocurrent generation and power conversion efficiency^5,6^. Specifically, protons covering the energy range of about 10–100 MeV and electrons covering the energy range of 1–0.11 MeV are extremely damaging to silicon because they have a high penetration depth and a high lattice interaction cross-Sects.^7,8^. Due to the cumulative emission of radiation, conventional C-Si solar cells at Medium Earth orbit (MEO) and at Geostationary Earth orbit (GEO) tend to lose 20–40% of power conversion efficiency over mission-relevant periods with a primary source of degradation being the decrease in V_oc_, J_sc_​, Fill Factor (FF), and Power Conversion Efficiency (PCE)^9,10^.

Radiation-hardened silicon solar cells are utilized along with a cover glass shield, redundant power architecture and conservative design margins, substantial radiation-induced performance degradation is observed over long periods of operational life^11,12^. It has been experimentally and in-field determined that traditional shielding techniques are effective in reducing low-energy particles and ionising dose effects. However, high-energy protons and electrons still cause displacement damage in the silicon lattice and result in a cumulative efficiency loss^13,14^. Consequently, even hardened silicon solar cells usually have power outputs which are measurably reduced with extended radiation exposure, which constrains their use in long-duration terrestrial photovoltaic missions. Furthermore, the traditional qualification and irradiation testing are limited to testing over a narrow range of fluence and short exposure durations over a small range of fluence and short exposures, which do not reflect the time-dependent degradation effects of accumulation of defects, interface instabilities, and gradual carrier lifetime degradation over mission-relevant periods^15,16^. The constraints have led to the creation of new materials and predictive modelling software to improve the long-term stability, radiation tolerance, and stability of performance of terrestrial photovoltaic solar cells.

Graphene has proven to be a promising solution to these shortcomings; a few of its promising features include its atomically thin two-dimensional structure, carrier mobility of 10^4^ cm^2^ V^−1^ s^−1^, optical transparency of 97% and its outstanding chemical as well as radiation stability. In combination with crystalline silicon, graphene results in a Schottky-type or passivated heterojunction that creates a built-in electric field at the interface, facilitating the separation of charge carriers and reducing losses due to surface recombination. Such a junction structure is beneficial for band alignment between graphene and silicon, reducing interface defect sensitivity and improving carrier collection efficiency, especially when the condition is defective under radiation^17,18^. Accordingly, the graphene silicon heterojunction devices provide an opportunity with regard to countering the radiation-based degradation processes that restrain the high-performance of traditional silicon photovoltaic devices in the long run.

Graphene is remarkably radiation-tolerant and shows minimal structural damage under experimental conditions, showing little or no structural damage as well as the low degradation in electrical conductivity following exposure to proton and electron fluences that are tens of times higher than 10¹¹-10¹^5^ cm^−1^^19,20^. Gr-Si heterojunction solar cells have demonstrated power conversion efficiencies up to about 18.8 per cent and high thermal stability and very little performance degradation when subjected to time and environmental limitations^21,22^. By incorporating graphene into devices in terrestrial-photovoltaic-grade PV systems, higher power-to-weight ratios, mechanical flexibility, and longer working lives of solar arrays mounted on satellites, rovers, and orbital platforms are possible, as summarised in Table 1^23^.

**Table 1:** Useful properties of graphene for solar cell efficiency^19–23^.

Property	Typical value / characteristic	Efficiency benefit
Electrical Conductivity	~ 10⁶ S/m	Low resistive losses, improved charge collection
Optical Transparency	~ 97.7% (monolayer)	Allows lighter to reach silicon absorber
Carrier Mobility	> 10⁴ cm^2^/V·s	Fast carrier transport, reduced recombination
Mechanical Strength	~ 1 TPa (Young’s modulus)	Maintains interface integrity under stress
Chemical Stability	High resistance to oxidation	Preserves material quality in harsh environments
Thickness	0.34 nm (monolayer)	Minimizes shading, enables tunneling contact
Work Function Tunability	4.5–4.8 eV (tunable via doping)	Enhances junction performance with silicon
Thermal Conductivity	3000–5000 W/m·K	Prevents thermal damage under irradiation
Radiation Hardness	Survives > 10¹⁴ particles-cm^−2^	Slows performance degradation in Terrestrial Photovoltaics

Despite extensive efforts to mitigate radiation-induced degradation in crystalline silicon solar cells through material engineering, shielding, and redundancy-based system design, a 20–40% long-term efficiency loss under mission-relevant radiation conditions remains unresolved Available experimental results are mostly limited by short degradation times and few such that are limited by fluence, although most models of degradation are based on empirical fits, or black box machine-learning methods, which are not physically interpretable and do not remember time-dependent defect evolution and interface degradation. Moreover, despite the promising performance of graphene-silicon heterojunction solar cells, which efficient, thermally stable and resistant to radiation, there exists an acute shortage of integrated structures that could quantitatively relate the radiation-induced physical degradation mechanisms to predictive changes in performance over operational lifetime. As a result, there is a marked lack of the development of physics-based, experimentally validated digital twin (DT) protocols that can effectively predict not only long-term performance and reliability of graphene-silicon photovoltaic systems in radiation-heavy systems on Earth.

The purpose of the research is to measure the radiation response and retention of the efficiency of graphene-silicon (Gr-Si) heterojunction solar cells at mission-relevant terrestrial photovoltaic operating conditions. Understanding the natural constraints of standalone laboratory irradiation experiments to measure time-dependent effects of degradation over time, a digital twin (DT) of the Gr-Si photovoltaic system with physics - driven modelling is created to recapitulate the dynamic nature of the actual device over its life cycle^24,25^. The concept of digital twins has become a revolutionary instrument in the engineering of terrestrial photovoltaic systems since it makes it possible to predictively maintain the system, monitor its performance constantly, and also adapt it to optical frameworks by integrating real-time experimental data and numerical data^26^. Applied to photovoltaic systems, DTs can model the ageing, defect accumulation of radiation sources, and behaviour of the device explicitly in terms of radiation damage, and predict device behaviour under different operational and environmental conditions^27,28^. This experiment has included Gr-Si solar cells that are exposed to a controlled amount of radiation, the cells are deeply characterised electrically, and then a physics-based computational model that involves radiation transport and degradation is developed. The predictions of the digital twins are then compared to the experimental results in order to determine the pathways of degradation and design optimisation approaches of the next-generation radiation-resilient terrestrial photovoltaic technology with a higher efficiency retention and a longer operation life.

## Materials and methods

### Fabrication of graphene-silicon heterojunction solar cells

Graphene-silicon (Gr-Si) Schottky heterojunction solar cells were built on a monocrystalline n-type silicon (300 μm) wafer with a resistivity of 1–10 Ω·cm. Wafers were first washed with a regular (Radio Corporation of America) RCA cleaning procedure, and then a short incubation in diluted hydrofluoric (HF) acid was done to remove the natural oxide layer and get a hydrogen-terminated surface^29^. In this process, the monolayer graphene was prepared on copper (Cu) foils through a chemical vapour deposition (CVD) of methane (CH_4_) and hydrogen (H_2_) at a growth temperature of approximately 1000 °C^30^. The transfer of the graphene layer on the silicon substrate was done through a wet transfer system that involved polymethyl methacrylate (PMMA). Aqueous acetone was used to remove residual PMMA, and the samples were then annealed in an argon-hydrogen environment to enhance bonding between the interfaces and minimise contamination caused by the transfer^31,32^. The layer of indium tin oxide (ITO), 50 nm thick, was deposited on the front side as a patterned shadow mask to define the active device. The back contact was created by applying a full-area layer of aluminium onto the backside of the wafer by means of electron-beam deposition and then thermal annealing at 400 °C to activate that contact and so that it behaves as an ohmic contact with a low resistance^33^.

The artificial Gr–Si solar cells had an architecture of vertical Schottky heterojunction, where the role of graphene was a transparent conductive electrode and Schottky contact, and the silicon substrate was the main light-absorbing layer and medium of charge- carrier generation^34^. The arrangement of this device offers greater optical transmittance, carrier transportation, and a preferable inherent electric field to effect photovoltaic activity^35,36^. There were twelve identical devices produced and kept in the nitrogen-filled enclosures before characterisation to ensure that minimum environmental degradation was experienced. The size of each device was an active area of 1 cm^2^, and it was produced in an ISO Class 5 cleanroom to ensure that it was not contaminated and that the interface oxidation was not unintended^37^. The Gr-Si devices were prepared by transferring monolayer films of graphene onto crystalline silicon wafers. The silicon substrates were pre-treated through regular RCA and then a slight thermal treatment to promote interfacial adhesion was performed before graphene transfer. The Graphene films were produced through the chemical vapour deposition (CVD) process and transferred to the silicon substrate with the help of polymer assistance wet transfer process, which maintains the structural integrity and continuity of the graphene layer.

**Fig. 1:**
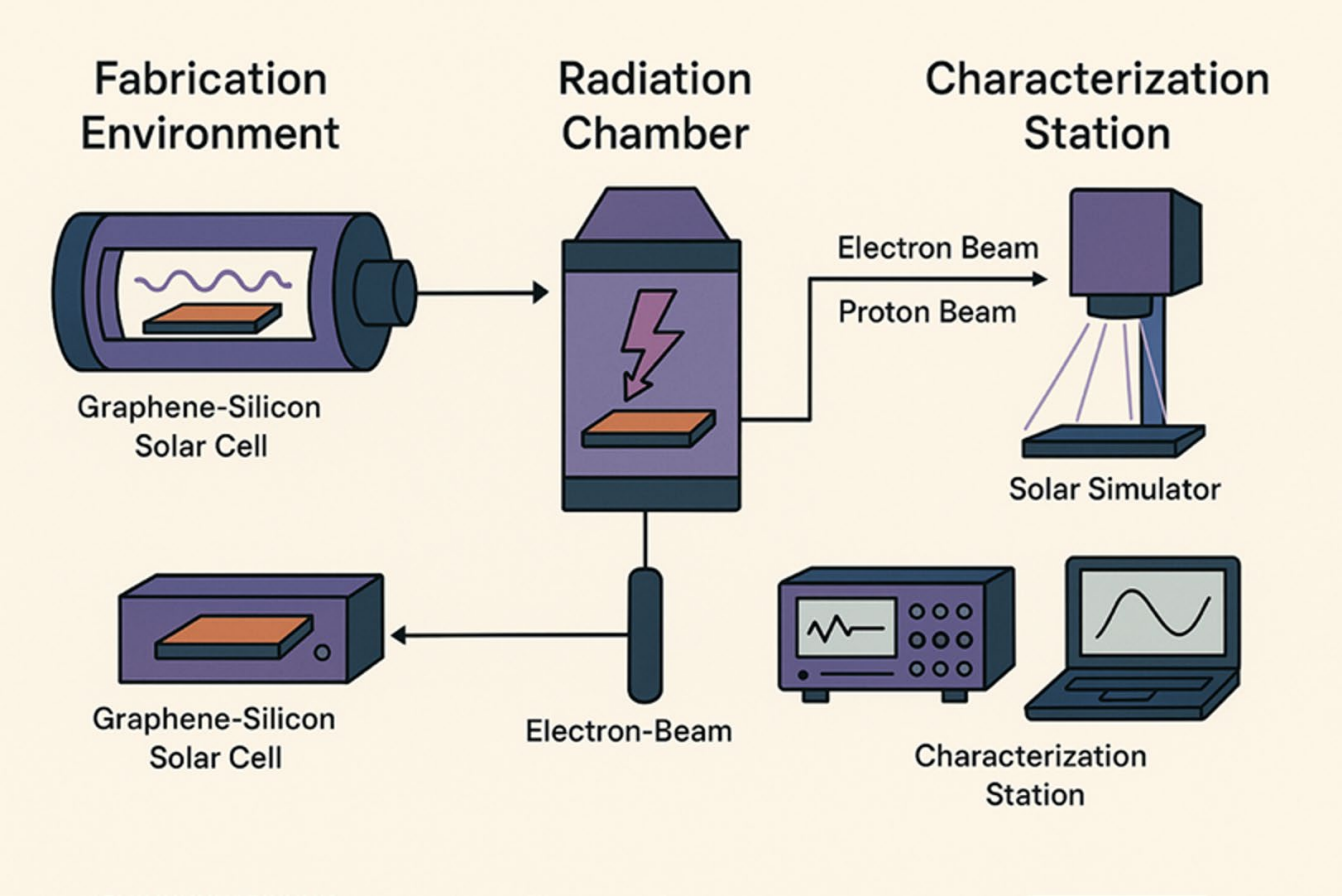
Schematic representation of the experimental workflow for evaluating graphene–silicon solar cells under irradiation conditions.

The fabrication, irradiation, and characterisation of the graphene-silicon (Gr- Si) solar cells were done using the experimental structure shown in Fig. 1. It starts with the monolayer graphene synthesis and transistors onto a clean crystalline silicon substrate to create Gr-Si heterojunction solar cell in controlled conditions. These artificial devices are then subjected to a special radiation chamber which aims at placing the devices in an environment similar to that of the terrestrial photovoltaic radiation where proton and electron beams of high energy are introduced upon the samples to mimic conditions in a mission environment. After the irradiation, transfer of the solar cells into a photovoltaic characterisation facility is done to undergo thorough electrical analysis. A calibrated solar simulator has the effect of replicating the standard solar spectrum to allow current voltage (I-V) measurements under standard test conditions. A source-measure unit with a data acquisition system is used to measure electrical performance parameters, such as V_oc_, J_sc_, and fill factor (FF), and power conversion efficiency (PCE). This combined experimental procedure of device fabrication and post-irradiation analysis offers a complete dataset of radiation-induced performance degradation and will facilitate the prediction of solar cell lifetime and performance dynamics under terrestrial photovoltaic operating conditions.

The X-ray diffraction (XRD) and Raman spectroscopy were used to assess the structural and material integrity of the samples before and after simulated irradiation. The XRD analysis was conducted to determine the crystallographic planes and determine the phase composition of the silicon substrate and the layer of graphene. The diffraction peaks of planes Si (111), (220) and (311), and the reflection of the graphene (002) were measured to confirm stability and crystalline integrity of the composite system structure. The graphene layer was characterised by the use of Raman spectroscopy, especially to determine the crystallinity and density of defects. Pre-irradiation spectra showed strong G and 2D bands, which attested to the high quality of the graphene, whereas post-irradiation measurements were tested with respect to change in peak intensity, position and D-band evolution to identify structural changes due to radiation.

Concurrently with experimental characterisation, a physics-informed semi-empirical digital twin (DT) model was created that has been designed to model the radiation-induced performance evolution of the graphene silicon (Gr-Si) solar cell under terrestrial photovoltaic operating conditions. The Large-scale Atomic/Molecular Massively Parallel Simulator (LAMMPS) was used to simulate the dynamics of molecular dynamics in order to capture the phenomena existing on interfaces and the degradation processes at the atomic scale. The structure development and atomic configuration were visualised and analysed with the help of Open Visualisation Tool (OVITO). The heterojunction model was made by laying down a sheet of monolayer graphene (100) onto a sheet of crystalline silicon (100), to make the Gr-Si interface. Properly chosen empirical potentials were used to model interatomic interactions to describe silicon lattice bonding, carbon-carbon interactions, and allow the formation of defects, interfacial reconstruction and radiation displacement effects to be simulated at the atomic scale.

Atomistic simulations using LAMMPS were done with a hybrid potential scheme with the AIREBO potential used to model C - C interactions in graphene (cut-off 2.0 Å to describe covalent interactions and extended cut-off 10.2 Å to describe van der Waals terms) and with the Embedded Atom Method (EAM) potential used to model Si -Si interactions in the silicon substrate. A simulation cell was made up of a crystalline slab of Si(100) of around 610 nm thickness containing around 20,000–40,000 atoms, and the monolayer sheet of graphene (lattice constant 2.46 Å) was added on its surface. The application of periodic boundary conditions in the x and y directions and a fixed boundary condition with a vacuum separation of the order of 2–3 nm in the z-direction was done to avoid spurious interaction of images. After some atomic configuration import, the conjugate gradient technique was used to minimise the energy until force tolerance dropped to less than 10^− 6^ eV/Å. The system was then left to stabilise at the NVT ensemble temperature of 300 K, under Nose-Hoover thermostat with a damping value of 0.1 ps. The time step used was 1 fs, and 10,000–50,000 steps (10–50 ps) were used depending on the process under analysis.

Thermal stress was simulated by increasing the temperature from 300 K to 800 K at a constant slow rate of about 5 K/ps, and then equilibrium was maintained at high temperature to assess interfacial stability and defect movement. A primary knock-on atom (PKA) model was used to model radiation damage, wherein a few selected Si or C atoms were given kinetic energies ranging from 20 eV-100 eV, larger than the displacement threshold energy of silicon (15 eV) and graphene (20–23 eV). This caused vacuity, interstitial defects and local lattice deformities. A system of tracking defect evolution was measured at multiple picosecond intervals and post-analysis was done using OVITO to measure coordination changes, vacancy density, and bond-length variation.

The atomic configurations produced using LAMMPS were exported to OVITO to analyse quantitatively the structural and defects. To measure the lattice disorder, vacancy formation, and interstitial defects at the graphene-silicon interface, common neighbour analysis (CNA) and the calculation of the coordination number were conducted. The number deviation (Δ CN ) and the vacancy concentration (Nv, in defects per nm^3^) were computed to trace the process of structural degradation induced by radiation. Elemental diffusion depth (generally in the range of 0.2–1.5 nm, depending on PKA energy) was determined by extracting one-dimensional composition profiles along the interface normal, and the size distribution and density evolution of defect clusters and changes in bond length (Δr, Å) and local strain (%) were determined over simulation timescales of 10–50 ps. These were atomistically obtained parameters that served as the quantitative inputs of the degradation used in calibration and validation of the digital twin model.

Radiation dose (fluence range 10^11^-10^14^ particles cm^− 1^), operational time (0-10^4^ hours equivalent), and a constant operating temperature of 40 °C were chosen as the main input variables on the digital twin. The performance metrics used are output performance namely; V_oc_, J_sc_, FF, and PCE. The controlled learning process used Random Forest Regression of 100–200 decision trees, which were being trained on an 80/20 train validation split. Model performance had a higher R^2^ of more than 0.96 with root mean square error (RMSE) less than 3% relative error in the prediction of PCE. Surface response maps were created to depict degradation of PCE as a result of radiation fluence and operating period and showed that under simulated conditions degradation rates would be approximately 0.5–1.2% per 10^12^ cm^−2^ of fluence increase. The digital twin can be used to predict performance and provide quantitative predictions of the graphene-silicon solar cell lifetime and predictive evaluation in radiation-rich terrestrial photovoltaic conditions through the combination of atomistic defect metrics with experimental I-V data.

### Radiation exposure setup

The radiation tolerance of the graphene-silicon (Gr-Si) solar cells, high-energy radiation that could simulate terrestrial conditions of photovoltaic operating was evaluated. Experiments on irradiation were performed in a high-vacuum chamber using calibrated sources of radiation and real-time dosimetry systems to be in control and monitor the fluence of the particles accurately. The irradiation was done on monoenergetic beam of 50 MeV by protons and 1 MeV by electrons. The choice of these particle energies was to simulate the non-thermal radiation population of solar proton events (SPEs) and trapped electrons fluxes typically experienced in low Earth orbit (LEO) and geostationary orbit (GEO)^38,39^. Electron irradiation was performed with a specialised accelerator and beamline that was optimised to produce a uniform beam delivery and controlled fluence. The solar cells had been irradiated across a large fluence range to obtain cumulative effects of degradation, with electron fluences of 10^12^–10^15^ particles·cm^−2^ and proton fluences of range 10^12^–10^14^ particles·cm^−2^. This has enabled the systematic evaluation of the radiation-induced performance degradation with respect to the type of particle, energy, and cumulative dose^40^.

Calibrated solid-state detectors were used to perform dosimetry, as the neighbouring test samples were subjected to a measurement uncertainty of a ± 5%, and a corresponding delivery of the fluence was accurate^41^. The beam uniformity in the 1 cm^2^ active device was ensured by a raster-scanning system and continuously checked by real-time feedback of Faraday cups, beam profile monitors, which leading to spatial non-uniformity ± 5%^42^. All the experiments of irradiation were carried out in a vacuum chamber of stainless steel with the vacuum evacuated to a low pressure of about 10^−3^ torr, emulating near-vacuum conditions on terrestrial photovoltaic operations^43^. The samples were placed on a thermally insulated stage and passive heat dissipation ensured that device temperatures were kept 20–25 °C during the irradiation to remove the effect of thermally induced degradation^44^. To ensure operational safety, the irradiation chamber was remotely controlled and shielded with lead and enclosed. The radiation exposures and safety procedures were applied based on the requirements of ISO 14,644 and International Atomic Energy Agency (IAEA) radiation safety standards^45^. The devices were then immediately sent to a fresh nitrogen atmosphere to avoid oxidation or degradation due to other ambient factors before characterisation after irradiation.

**Fig. 2:**
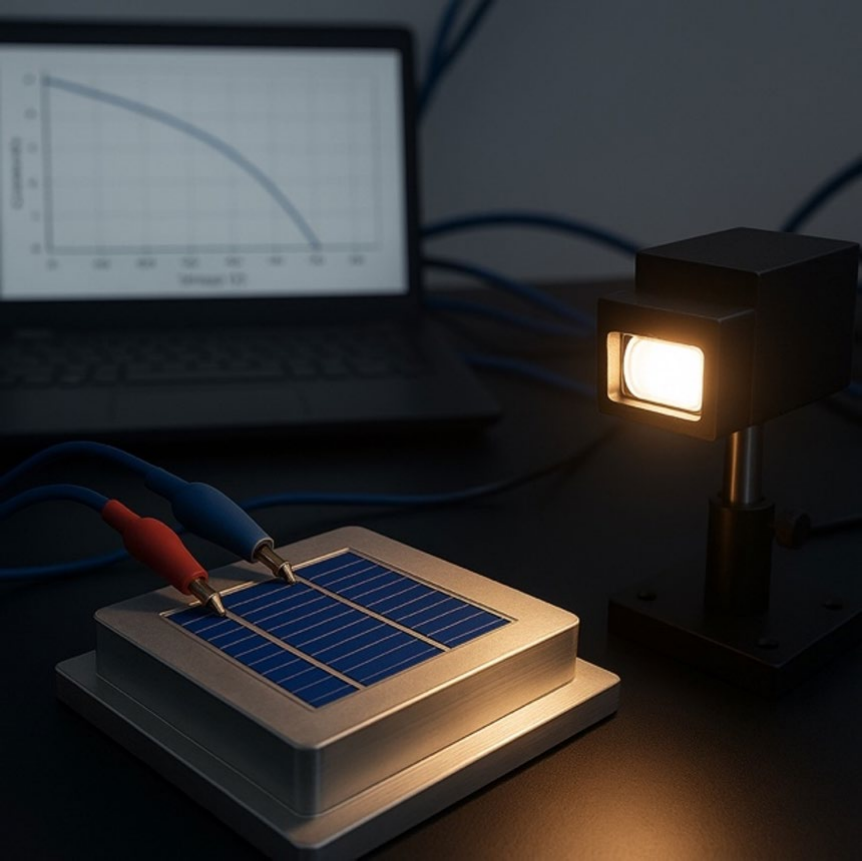
Experimental setup for photovoltaic performance testing of a graphene–silicon solar cell under controlled illumination.

Figure 2 represents the experimental designed to determine the performance of graphene silicon (Gr-Si) solar cells in simulated terrestrial conditions of photovoltaic operations. The system consists of a calibrated solar simulator, which provides a controlled light equivalent to the standard solar spectrum and, therefore, can be used to perform accurate photovoltaic testing. The solar cell is fixed on a metallic sample holder and real-time measurement of current voltage (I-V) characteristics were achieved by electrically contacting the solar cell with probes or crocodile clips. This device is designed to simulate the photovoltaic environment conditions of the Earth, and therefore, a vacuum and radiation chamber is used which enables the controlled exposure of the device to electron and proton irradiation and thermal environment changes. The sheets of the graphene layer are examined with a Raman spectrometer prior to irradiation and after it to evaluate the crystallinity and the formation of defects. Electrical performance parameters, such as V_oc_, J_sc_, FF, and PCE, are derived out of I-V curves presented using a computer-based data acquisition interface. This combined experimental system allows characterisation of electrical and structural simultaneously and offers this type of experimentation the validation required to simulate degradation and the evolution of performance with digital twins^46^.

### Characterisation techniques

To Assess the radiation induced impact on graphene silicon (Gr-Si) solar cells, post irradiation electrical, optical and structural characterisations were carried out extensively. Electrical performance was measured in the presence of a simulated sun in conditions of AM1.5G with an irradiance of 1000 Wm^−2^ coupled to a Keithley 2400 source-measure unit used to measure current-voltage (I-V) performance. The main photovoltaic parameters such as V_oc_, J_sc_, FF, and PCE were obtained prior to irradiation and following the irradiation to measure the rate of degradation. External quantum efficiency (EQE) measurements were performed at the wavelength between 300 and1100 nm in a monochromator based system using shutter control and lock-in amplification. The EQE analysis facilitated wavelength-yielding analysis of carrier generation and collection proficiency, which gave insight into radiation-induced losses, more especially in the near-infrared region corresponding to the silicon absorber^47^.

A 532 nm excitation laser was used in conducting Raman spectroscopy to determine radiation-induced structural changes in the graphene layer. The quantitative evaluation of defect generation was done through observing the change in the intensity ratio of the D band (~ 1350 cm⁻¹) to the G band (~ 1580 cm⁻¹) where the increment in the ID/IG ratio was the measure of the radiation induced disorder and lattice damage^48^. After and before irradiation, Raman spectra were measured, compared, and analysed to estimate the change in quantities of peak location, full width at half maximum (FWHM), and ratios of intensities, which are used to understand the dynamics of graphene defect density and network stability.

High-resolution scanning electron microscopy (SEM) was used to analyse surface morphology and radiation-induced physical damage such as crack formation, delamination and sputtering of particles. The integrity of the graphene and silicon interface at the nanoscale was examined by transmission electron microscopy (TEM) to observe the presence or absence of interfacial voids, atomic-level defects or the presence or absence of interdiffusion after irradiation^49^. Frequency response spectroscopy FRS measurements were made at frequencies 1 Hz to 1 MHz, using a frequency response analyser. An equivalent circuit model was used to analyse Nyquist and Bode plots to determine the series resistance (Rs), recombination resistance (Rrec) and junction capacitance enabling a quantification of carrier transport, recombination processes as well as interface degradation during radiation exposure. Variations in surface energy after the irradiation were measured by means of contact angle measurements (at rest) and the change in wettability was related to radiation effects of surface modification and contamination. All of the characterisation measurements were taken at controlled temperature of 22 ± 1 °C to avoid thermal drift and have reproducible characterisation parameters.

## Results and discussion

### Micro and nano structural analysis

The discovery of the effect of graphene on the performance and radiation strength of silicon-based solar cells was done through a systematic workflow that included the fabrication of the device, baseline performance analysis, radiation exposure and post-irradiation characterisation. Monolayer graphene was made on copper foil through chemical vapour deposition and transferred to cleaned light doped n-type crystalline silicon wafers. The graphene layer was an effective transparent conductor and a front conductive electrode at the same time. Contact to the graphene was made through the deposition of indium tin oxide (ITO) by magnetron sputtering and a full-area aluminium deposit was made to the rear surface of the silicon substrate to create a low-resistance ohmic contact. After the step of fabrication, all devices underwent thermal annealing to enhance the quality of interfacial contacts and eliminate residual contaminants, which were introduced in the fabrication of the devices, as well as, to measure the effect of graphene on the performance and radiation robustness of silicon-based solar cells.

The study of the effect of graphene on the operation and radiation strength of silicon-based solar cells in a systematic workflow included the fabrication of the devices, the baseline performance, radiation exposure, and characterisation after irradiation. Monolayer graphene was produced on copper foil using chemical vapour deposition and after that transferred onto clean and lightly doped n-type crystalline silicon wafers. The graphene layer was also used as a highly transparent window as a conductive electrode on the front side. ITO contacts were created by magnetron sputtering onto the layer of graphene and a complete-area layer of aluminium was created on the back side of the silicon substrate to create a low-resistance ohmic contact. After the fabrication, all machines were thermally annealed to enhance the quality of interfacial contact and also eliminate some residual contaminant that had been deposited on the machines through the process of graphene transfer.

**Fig. 3:**
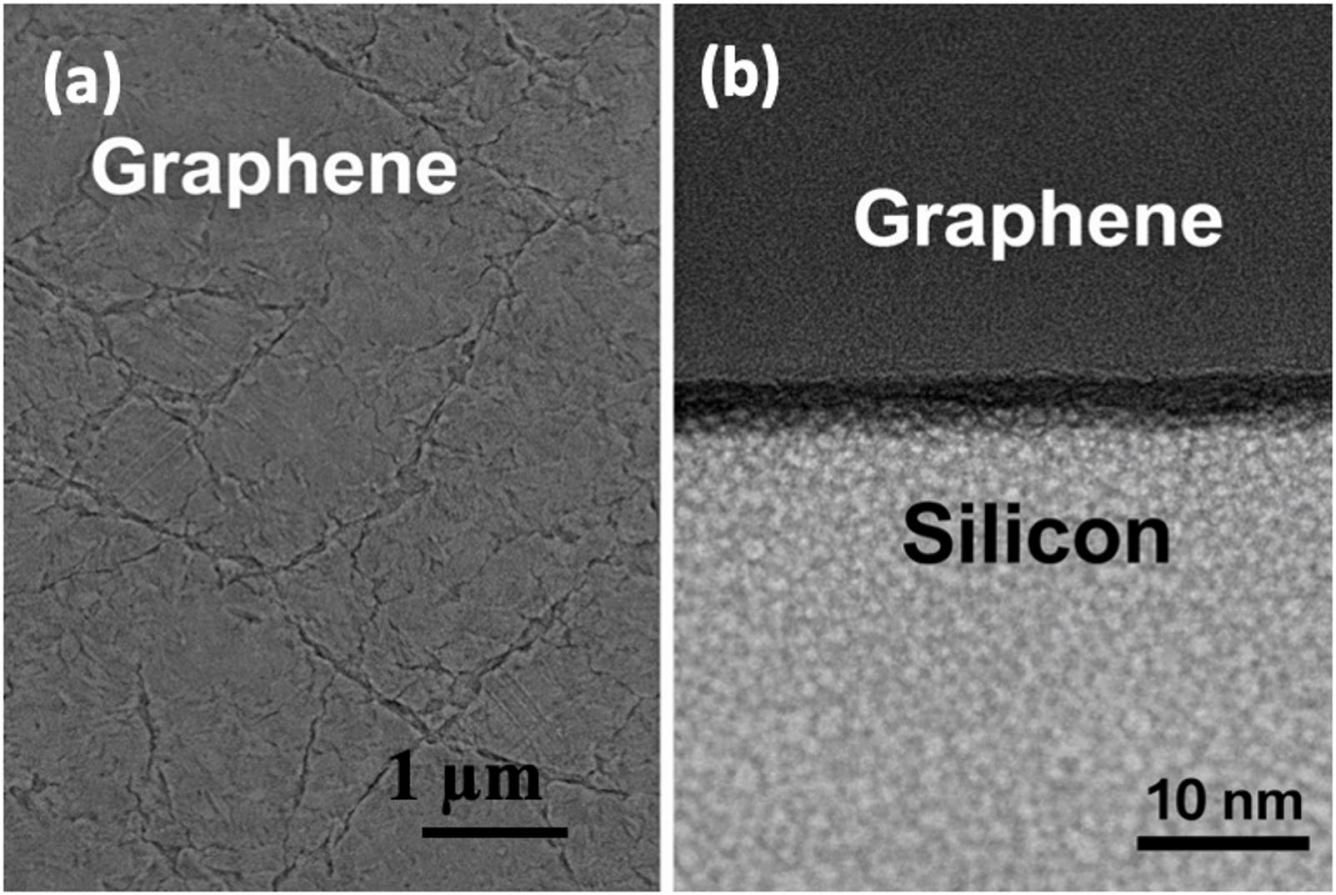
(**a**) Scanning Electron Microscopy (SEM) image showing the surface morphology of the graphene layer, with visible wrinkle-like features and grain boundaries indicative of transferred monolayer (**b**) Cross-sectional Transmission Electron Microscopy (TEM) image of the graphene–silicon heterojunction.

Figure 3 shows the microstructure of the graphene and silicon heterojunction in the form of scanning electron microscopy (SEM) and transmission electron microscopy (TEM). Figure 3a, which is the SEM picture of the transfers in the graphene film, reveals that the surface of the graphene film consists of a network of wrinkle-like features as well as distinct grain boundaries with lateral dimensions measured by a matter of hundreds of nanometers to a few micrometers or so. These morphological characteristics are common with monolayer or few-layers graphene that has been transferred off copper substrates and are credited to the remaining thermal stress, energy mismatch, and localized overlap in the wet-transfer process. Although wrinkles are present, the layer of graphene has continuous coverage over the electrode surface, which means that the device does not contain large-scale dislocations that are capable of slowing down the lateral carrier transport.

Figure 3b shows that the cross-sectional TEM image provides a sharp and clear interface between the layer of graphene and the crystalline silicon substrate. The graphene is viewed as a smooth dark contrasting layer and has a thickness that is estimated to be less than 1 nm, which is monolayer graphene. The sub-strain silicon has a highly ranked crystalline structure and no amorphous areas, voids or interfacial dissociation are seen at the measurement determination limit. The lack of interfaces separating the graphene and silicon and the conformal contact between them signify a strong adhesive interfacial bond, which is key in the separation and conduction of charges. This type of clean and continuous heterojunction reduces the interface-induced recombination pathways and enables stable Schottky junction behaviour in the graphene silicon solar cell.

**Fig. 4:**
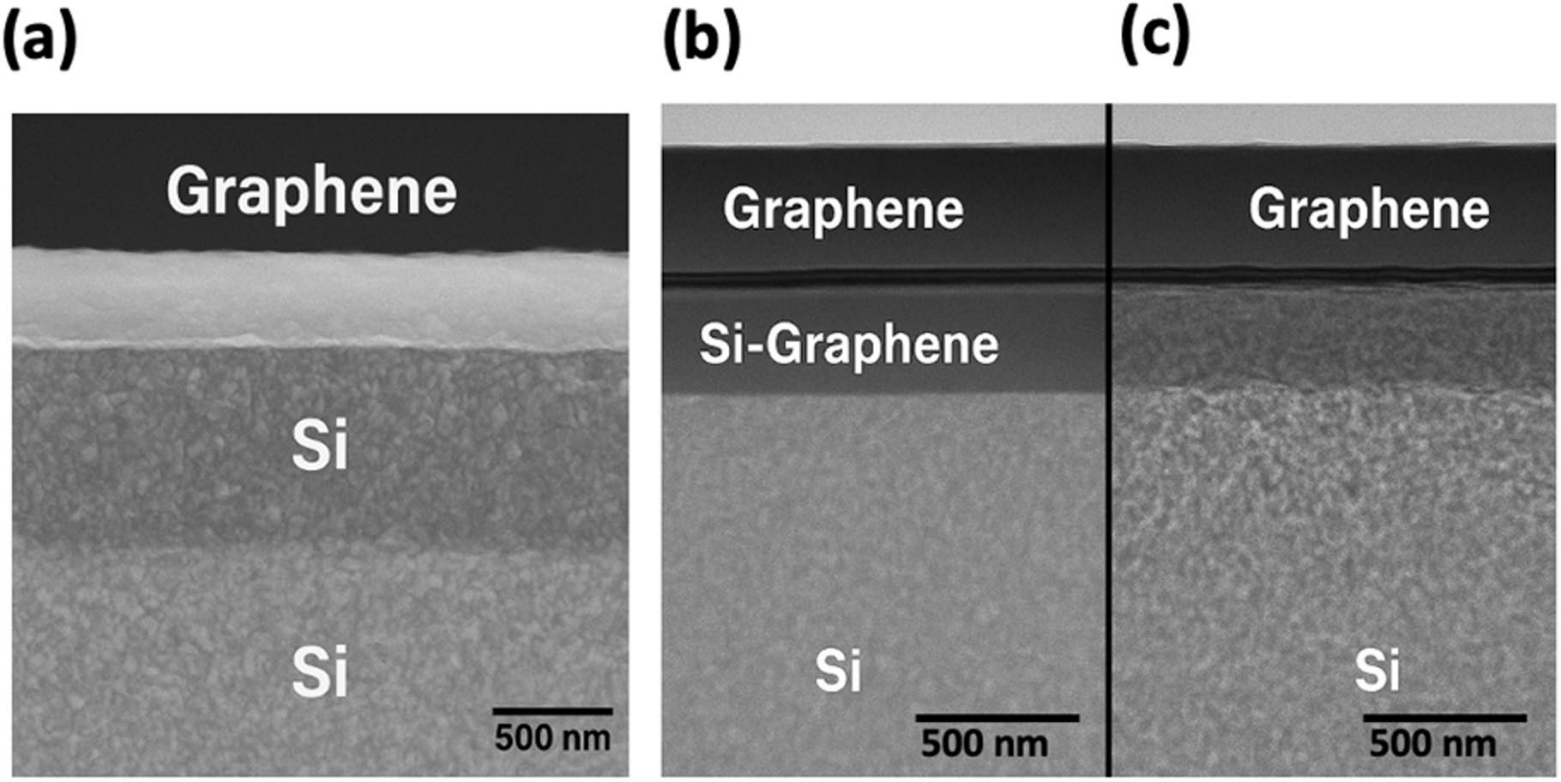
Cross-sectional Transmission Electron Microscopy (TEM) images of graphene–silicon heterojunctions (**a**) showing a continuous graphene film on top of the crystalline silicon substrate with a uniform interface, confirming successful layer transfer. (**b**) Higher magnification reveals a distinct interfacial region labelled as “Si–Graphene,” indicating a possible reaction or transition layer formed during processing or post-treatment. (**c**) A structurally intact graphene–Si interface without interlayer distortion, highlighting good adhesion and minimal disruption at the nanoscale.

Figure 4 is a high-resolution transmission electron microscopy (HRTEM) image of the graphene-silicon interface, which gives nanoscale information of the interfacial structure and integrity of graphene. Figure 4a indicates that there is a uniform distribution of a thin and continuous layer of graphene spread over the crystalline silicon substrate. The interface looks clean and properly aligned, and no delamination, void, or structural discontinuities can be seen within the resolution range of the measurement experiment, which confirms that the transfer of graphene and the intense adhesion between the two surfaces occurred. The graphene layer is uniform throughout the imaged area, which is very important when it comes to uniformity of electronic contact and stability of carrier transport in device applications.

A closer look at the graphene-silicon interface in Fig. 4b indicates that there is a differentiated interfacial area that is termed as a Si-graphene boundary layer. This area is probably formed by high-temperature processing steps or atomic-level diffusion during processing or post-annealing. The contrast change that has been seen indicates that there is little interfacial mixing or the potential to create SiC bonding between the interfaces that can locally alter band alignment and the junction transportation properties. These interfacial characteristics are important in defining the efficiency of charge transfer, the intrinsic strength of the built-in electric field, and the stability of operation of the graphene silicon heterojunction devices and radiative stability when subjected to operational and radiative demands. Figure 4 is a high-resolution transmission electron microscopy (HRTEM) image of the graphene-silicon interface, which gives nanoscale information of on the interfacial structure and integrity of graphene. Figure 4a indicates that there is a uniform distribution of a thin and continuous layer of graphene spread over the crystalline silicon substrate. The interface looks clean and properly aligned, and no delamination, void, or structural discontinuities can be seen within the resolution range of the measurement experiment, which confirms that the transfer of graphene and the intense adhesion between the two surfaces occurred. The graphene layer is uniform throughout the imaged area, which is very important when it comes to the uniformity of electronic contact and stability of carrier transport in device applications.

A closer look at the graphene-silicon interface in Fig. 4b indicates that there is a differentiated interfacial area that is termed as a Si-graphene boundary layer. This area is probably formed by high-temperature processing steps or atomic-level diffusion during processing or post-annealing. The contrast change that has been seen indicates that there is little interfacial mixing or the potential to create SiC bonding between the interfaces that can locally alter band alignment and the junction transportation properties. These interfacial characteristics are important in defining the efficiency of charge transfer, the intrinsic strength of the built-in electric field, and the stability of operation of the graphene silicon heterojunction devices and radiative stability when subjected to operational and radiative demands.

When magnified e.g. (Fig. 4c) the graphene-silicon heterojunction has a structurally sharp interface with no visible pores nor lattice deformation or interfacial boundaries in the resolution of the HRTEM image. The layer of graphene is conformally bonded to the silicon surface, which means that it is strongly bonded by van der Waals forces and, perhaps, by localised silicon-carbon bonding created during processing. The lack of interfacial interfaces or delamination will tend to eliminate interface-mediated recombination mechanisms and ensure the efficient charge transfer across the junction, which will sustain constant electrical performance.

SEM and TEM findings demonstrate that the structural integrity and the quality of the graphene-silicon heterojunction are high. The existence of a clean, continuous interface between and after processing is evidence of its efficacy as a usable Schottky heterojunction in radiation-tolerant photovoltaic devices. These microstructural observations confirm the model of geometric assumptions and interface stability parameters used in the degradation model and digital twin model, enhancing the correlation between the simulated prediction and experimental results of the device behaviour of radiation-stress tests. When magnified e.g. (Fig. 4c) the graphene-silicon heterojunction has a structurally sharp interface with no visible pores nor lattice deformation or interfacial boundaries in the resolution of the HRTEM image. The layer of graphene is conformally bonded to the silicon surface, which means that it is strongly bonded by van der Waals forces and, perhaps, by localised silicon-carbon bonding created during processing. The lack of interfacial interfaces or delamination will tend to eliminate interface-mediated recombination mechanisms and ensure the efficient charge transfer across the junction, which will sustain constant electrical performance.

Collectively, the SEM and TEM findings demonstrate that the structural integrity and the quality of the graphene-silicon heterojunction are high. The existence of a clean, continuous interface between and after processing is evidence of its efficacy as a usable Schottky heterojunction in radiation-tolerant photovoltaic devices. These microstructural observations confirm the model of geometric assumptions and interface stability parameters used in the degradation model and digital twin model, enhancing the correlation between the simulated prediction and experimental results of the device behaviour of radiation-stress tests.

### XRD and Raman spectra analysis

**Fig. 5:**
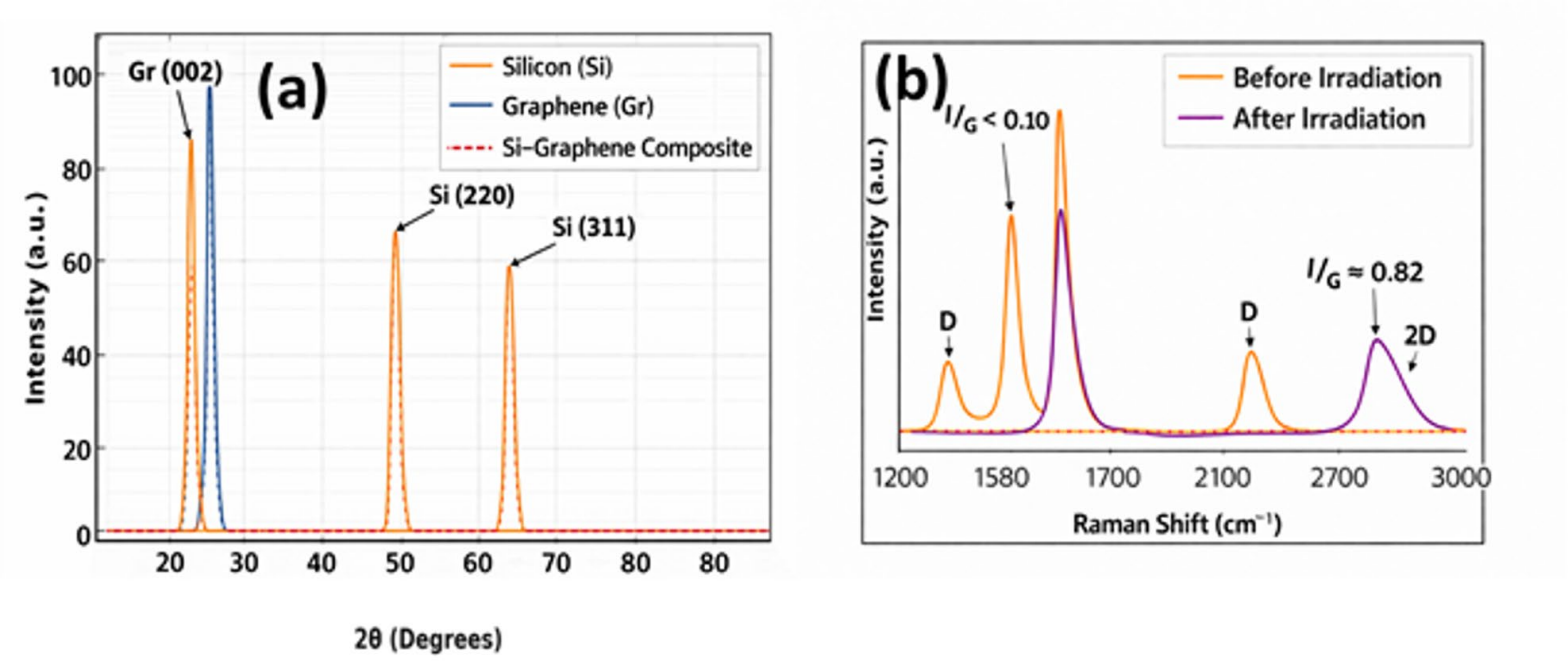
(**a**) X-ray Diffraction (XRD) patterns showing characteristic peaks of pure silicon (Si), graphene (Gr), and the Si–graphene composite. (b) Raman spectra of graphene before and after irradiation. Pre-irradiation spectra show sharp G (~ 1580 cm⁻¹) and 2D (~ 2700 cm⁻¹) bands, indicating high crystallinity. Post-irradiation, the emergence of a D band (~ 1350 cm⁻¹) and reduction in 2D intensity reveal defect formation and lattice disorder due to radiation exposure.

Figure 5a presents the structural characterization of the graphene- silicon heterojunction obtained through X-ray diffraction (XRD). The pattern of diffraction attests the fact that there are specific crystalline phases that relate to silicon and graphene. The silicon sample has typical peaks of 2θ ≈ 28.4° (Si (111)), 47.3° (Si (220)), and 56.1° (Si (311)) that are similar to crystalline silicon. The crystalline nature of the graphene layer is also confirmed by a strong peak at a 2 literally 2θ ≈ 26.5°, typical of the plane of graphene (002). The appearance of all these diffraction characteristics, without any visible peak shifting or broadening, is evidence of successful formation of the graphene-silicon heterostructure without causing either of the materials to undergo any significant lattice distortion.

The Raman spectra of the graphene samples just before and after proton and electron irradiation are shown in Figure 5b. Before irradiation, the G band (~1580 cm^-1^) and the 2D band (~2700 cm ^-1^) constitute the spectrum with the insignificant D band, which means that the crystalline order is high, and the defect concentration is low. Following the process of irradiation, a separate D band appears at a position of approximately ~1350 cm⁻^1^.On a quantitative basis irradiation results in the expansion of the ratio of the D- to G-intensities (ID /IG ) which directly measures the number of defects created by exposure to high-energy particles. These findings indicate that radiation causes quantifiable disorder to the graphene lattice and does not eliminate its overall crystalline structure.

These findings prove that even though the graphene-silicon heterostructure is not damaged on the macroscopic level in terms of its general crystalline order, irradiation produces localised defects on the atomic scale, which cannot be directly observed using bulk structural probes on their own. The fact that such point defects and lattice disorder exist demonstrates the significance of considering the explicit models of defect formation and evolution in the estimation of degradation due to radiation. The changes in the spectroscopy that are observed collectively show the effect of the terrestrial photovoltaic-like radiation on the structure of the materials and highlight the importance of predictive and time-resolved monitoring with the aid of a digital twin framework. The digital twin can capture the gradual degradation of the electronic properties by using experimentally gained metrics of defects and is capable of determining the efficiency loss due to radiation which can be precisely predicted in all cases, which confirms its ability to predict the long-term performance of the device under radiation conditions.

### Atom probe tomography (APT) analysis

**Fig. 6:**
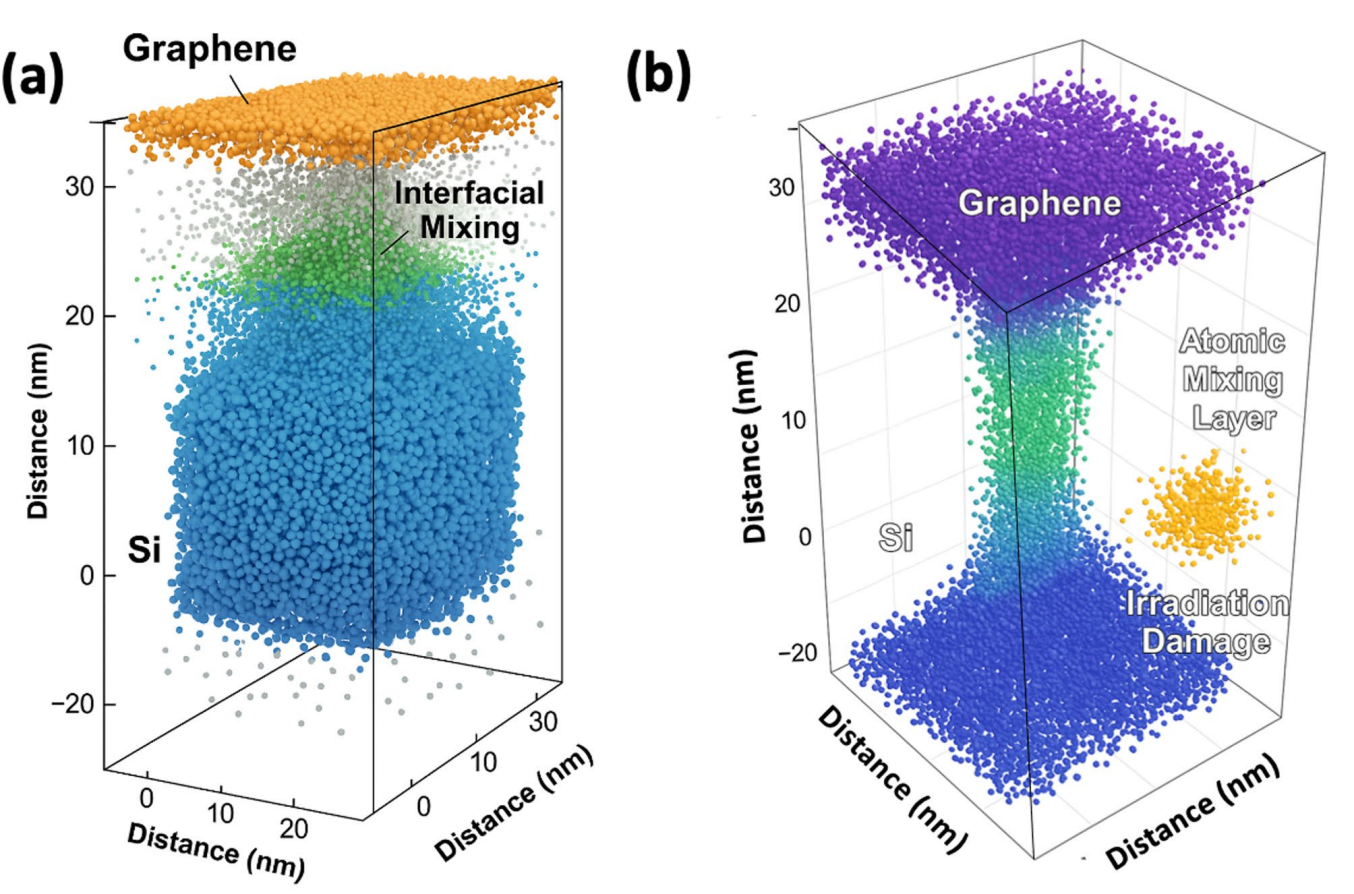
Three dimensional three dimensional atom probe tomography (APT) images of the graphene-silicon heterojunction. (**a**) Pre-irradiation condition, which exhibits a sharp and chemically discontinuous interface between graphene and silicon with a thin interfacial mixing layer, which is a sign of little atomic interdiffusion. (**b**) Post-irradiation, where the layer of graphene is continuous, but massive interfacial broadening is evident, which creates a broadened atomic mixing layer. A cluster of defects in the silicon region under the influence of localized irradiation is observed, which is in line with the atomic displacement and defect formation under high particle energy irradiation.

Atom probe tomography (APT) was incorporated within the digital twin model to gain atomistic-level information about the graphene-silicon heterojunction and to determine the prevailing degradation pathways. Figure 6a depicts a 3D representation of the APT reconstruction of the spatial distribution of individual atoms in the heterointerface. The graphical paper, which has been made to look orange, is so distinctly visible against the silicon material, which is depicted in blue. An interfacial mixing zone, which is characterised by a clear boundary, is observed between these two regions, with carbon and silicon atoms being the main elements, with a lesser contribution of oxygen and phosphorus.

Interfacial mixing is limited to a thickness of about 5–7 nm which means the lack of atomic interdiffusion. This thin interface layer is probably connected with the first graphene to silicon contact and the remaining species that were presented during the transference and processing methods. Significantly, the layer of graphene is continuous, planar and chemically stable throughout the volume under analysis, indicating that there is a high level of chemical compatibility as well as mechanical stability at the interface. The interfacial details observed by APT at an atomic scale can be attributed to the general patterns of the mechanisms of degradation predicted by the digital twin, especially the ones associated with diffusion based on thermal effects, interface stability, and the development of defects under radiation. This high consistency supports the fact that the mechanisms of interfacial degradation are physically modelled by the digital twin.

In Fig. 6b, the atom probe tomography (APT) reconstruction of the graphene silicon heterojunction is presented after the exposure to a high radiation fluence (≥ 10¹⁴ particles cm^−2^). Although the graphene layer can be distinguished and continuous, the region between them changes to a severe broadening of the atomic mixing in comparison to the pristine state. The quantitative analysis shows that the interfacial mixing thickness increases past the first 5–7 nm depths in line with radiation-enhanced atomic mobility and defect-assisted diffusion mechanisms. Redistribution of carbon and silicon atoms across the interface is an indication that, high-energy particle irradiation is a favourable contributor to displacement cascades, which facilitates intermixing at existing defect locations.

The interfacial changes that occur by radiation are credited to the concentration of point defects such as vacancies and interstitials and their consequent clustering and segregation at the heterojunction. Such aggregation of defects which are here denoted by irradiation damage is a typical response to displacement damage when exposed to high-fluence irradiation. Also, local lattice distortion and minimal interfacial warping can be seen around the interface edges which implies the existence of irradiation-induced stress and potential thermo-mechanical effects. These characteristics imply that strain is accumulated on a small scale but not at a macro level, which is a confirmation that an interface is also mechanically stable and subjected to a controlled structural modification in the presence of extreme radiation conditions.

**Fig. 7:**
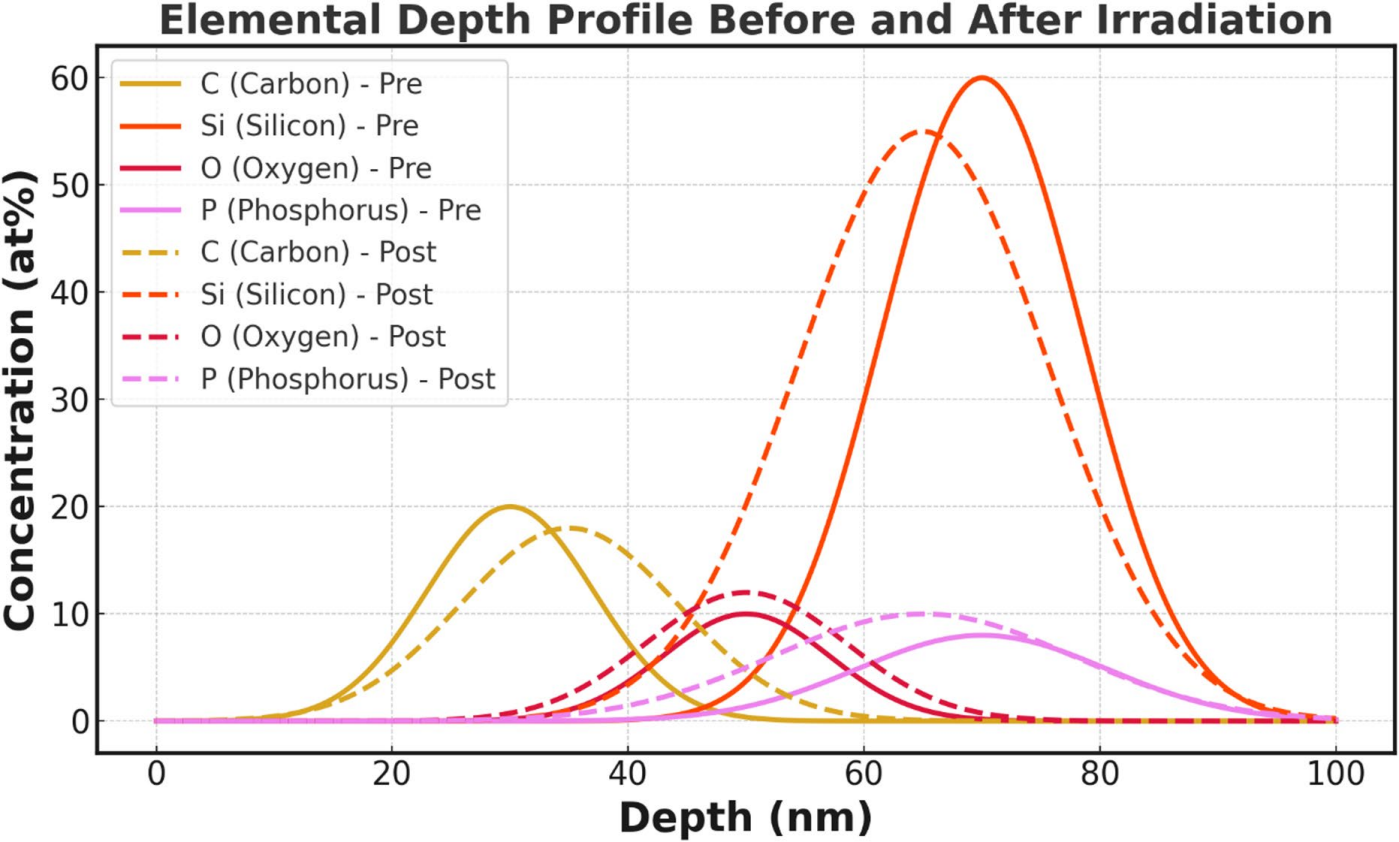
Atom probe tomography (APT) results of elemental depth concentration of carbon (C), silicon (Si), oxygen (O), and phosphorus (P) at pre-irradiation and post-irradiation. The profiles of pre-irradiation (solid lines) have well-separated elemental peeks that are indicative of a chemically abrupt interface, whereas the post-irradiation profiles (dashed lines) have broad distributions indicative of radiation induced interdiffusion and interface mixing.

Figure 7 shows results of atom probe tomography (APT) of 1D concentration depth profiles of carbon (C), silicon (Si), oxygen (O) and phosphorus (P) at the start and end of irradiation. The elemental distributions show clear and spatially localised peaks of the graphene layer (C-rich region) and bulk silicon substrate (Si-rich region) and a thin interfacial region between the two in which oxygen and phosphorus can be detected in trace levels before irradiation (solid lines). The case of acute edges between these areas demonstrates a chemically sharp graphene-silicon surface with slight interdiffusion in the pure state.

After irradiation (dashed lines) the elemental profiles exhibit highly broadened profiles across the interface. The carbon concentration maximum becomes smaller and further penetrates into the silicon area, which demonstrates that diffusion of carbon atoms of the graphene layer and substrate under the influence of radiation increases. At the same time, the silicon contour is expanding towards the surface along with the oxygen concentration beyond the surface and toward the near-interface that is representative of the bi-directional atomic mixing. After irradiation, oxygen and phosphorus show higher concentrations in the interfacial region and a broader spatial distribution of the elements indicating that high-energy particle exposure facilitates diffusion involving defects and impurity segregation. The increased oxygen level can also be connected with the radiation-initiated oxidation or the relocation of remaining contaminants.

The relocation of these components ascertains the creation of a radiation-induced interfacial mixing layer, which is in line with the structural corrosion of the TEM and three-dimensional APT reconstructions. This chemically expanded interface is associated with the observed decreases in open-circuit voltage and fill factor, and the radiation-induced interfacial defect and compositional disorder has a negative impact on charge transport and recombination processes. Generally, the findings indicate that although the graphene-silicon interface is chemically sharp and stable before irradiation, radiation exposure mainly damages the junction by interfacial broadening and defect creation instead of on a large-scale delamination or bulk phase transformation.

### Radiation tolerance performance

**Fig. 8:**
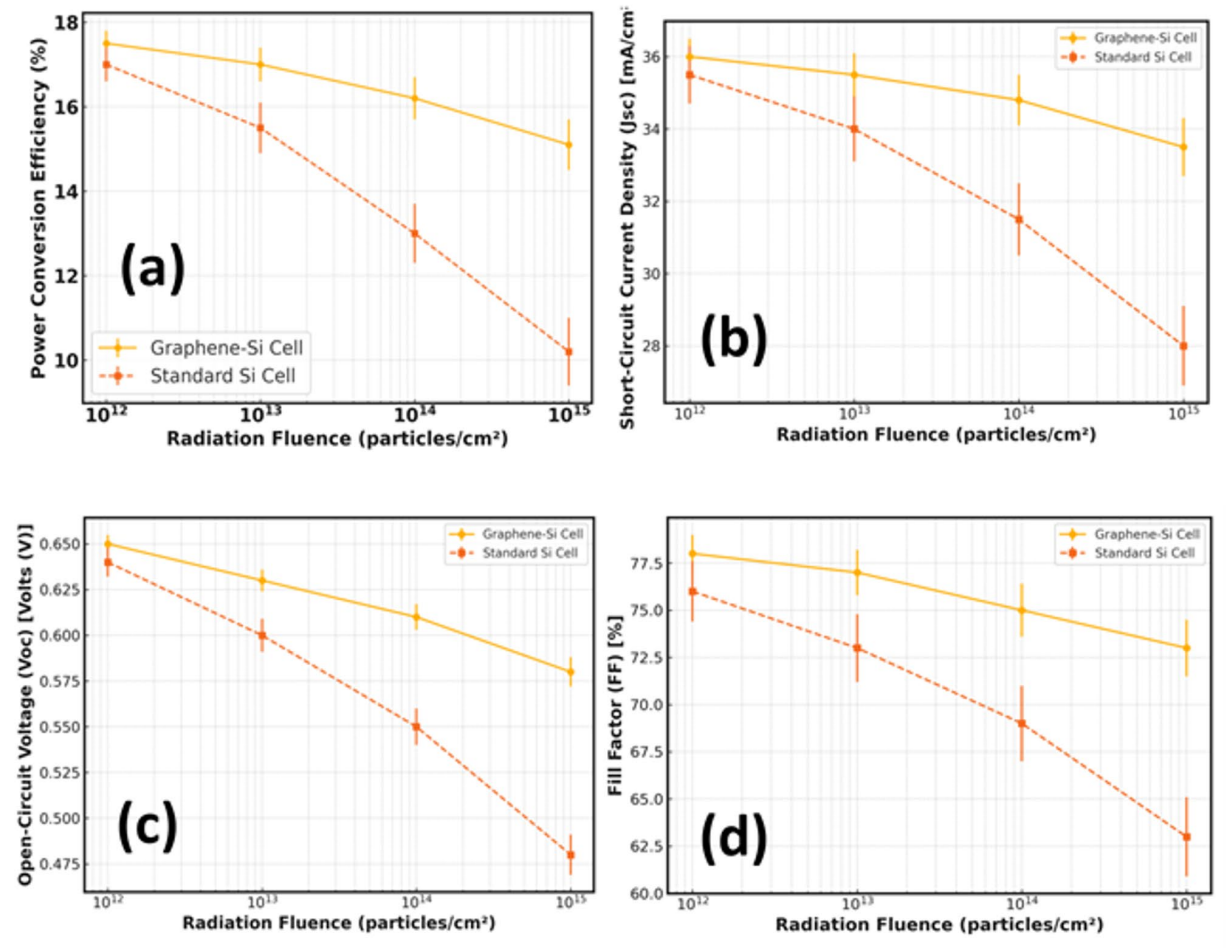
Comparison of radiation-induced photovoltaic performance degradation in Gr–Si and conventional silicon (Si) solar cells with increasing radiation fluence: **(a)** power conversion efficiency (PCE), **(b)** J_sc_, **(c)** V_oc_, demonstrating slower degradation in the Gr–Si device, and **(d)** FF, showing a monotonic decrease for both cell types.

In Fig. 8a, there is a comparison of the radiation-induced degradation of the V_oc_, J_sc_, FF, and PCE of graphene-silicon (Gr-Si) and traditional crystalline silicon (Si) solar cells with increasing radiation fluence at 10^12^ to 10^15^ particles·cm^−2^. The Gr-Si devices are much more stable across the whole range of fluence. The PCE of the Gr-Si cells at the maximum fluence of 10^15^ particles·cm^−2^, is reduced by approximately 2.3 per cent compared to that of the pristine. Conversely, the traditional Si cells exhibit a significant efficiency reduction, and the PCE drops to less than 17.5%, and resulting in a relative loss is over 11%. This comparison already shows the enhancement of radiation tolerance by the graphene layer.

Figure 8b indicates the J_sc_ changes with the radiation fluence. In both types of devices, J_sc_ monotonically decreases as the fluence increases, because of the creation of radiation-induced recombination centres, and shorter minority carrier lifetimes. Nonetheless, the degradation rate is much reduced when it comes to Gr-Si devices. At 10^15^ particles·cm^−2^, the Gr-Si cells can retain a J_sc_ of about 33 mA·cm^−2^, and the traditional Si cells reduce to about 28 mA·cm^−2^. This minimised current loss which means that more efficient carrier collection and tolerance to defects in the presence of graphene.

The trends of V_oc_ as displayed in Fig. 8c also indicate that the Gr–Si solar cells are more radiation resilient. The V_oc_ of the Gr-Si devices at high-fluence irradiation values is near 0.60 V whereas the V_oc_ of the conventional Si cells is less than 0.50 V. The enhanced V_oc_ stability is due to the decreased interface recombination and the maintained band positioning at the graphene silicon junction which softens the effects of the radiation induced defects. All in all, the integrated electrical parameters prove that the integration of graphene can highly reduce the radiation-induced performance loss of silicon solar cells.

Figure 8d demonstrates the change in fill factor (FF) versus radiation fluence of Gr–Si and standard silicon (Si) solar cells. The Gr-Si devices have a rather low FF degradation, which reduces between 78% in the pure form to about 74% at the maximum fluence of 10^15^ particles·cm^−2^, which is a relative decrease of about 5%. The traditional Si cells, on the contrary, exhibit a significantly smaller FF decrease, decreasing between ~ 76% to ~ 62%,, or a relative loss of about 18%. The enhanced FF retention in Gr-Si cells shows reduced radiation induced series resistance changes and contact and junction properties in high-fluence irradiation.

The integrated results of PCE, J_sc_, V_oc_, and FF, in general, indicate that the integration of graphene in silicon solar cells significantly improves the radiation tolerance of these cells, allowing them to retain electrical functioning even in difficult situations where extreme particles are exposed. Also the fact that graphene is a strong charge-collection layer. Graphene has a high mobility and radiation stability, which conserves the carrier extraction pathways and the Schottky barrier between the graphene and silicon surface traps efficient charge separation despite the displacement damage in the silicon bulk. This was the reason why V_oc_, J_sc_, and FF degraded more slowly when under high-fluence irradiation. These findings make graphene-silicon solar cells a usable and viable photovoltaic technology to be used in terrestrial photovoltaic projects, where long-term exposure to high-energy particles is a serious challenge to the long-term reliability of the devices.

### Digital twin framework

The terrestrial photovoltaic environment simulation Digital twin simulations were carried out by combining experimentally obtained data with artificially generated data to simulate long-term device behaviour. The main aim was to estimate the changes in the V_oc_, J_sc_, FF, and PCE in relation to radiation exposure and working time in a quantitative manner. Since obtaining long-period, high-fluence experimental data is practically limited, a reference dataset was built upon the basis of the trends of degradation provided in the previous radiation studies and confirmed experimental data. The radiation dose simulated was between 10⁴ and 10⁷ Gy, whereas the working hours were as long as 200 h. All the simulations were maintained in a constant operating temperature of 40 °C to separate the dose effects and the effects of time of exposure of radiation.

Supervised machine learning using the Random Forest Regression algorithm was used to predict the electrical performance metrics of the input parameters (radiation dose, exposure time, and temperature) to develop a map. The reason why this model was chosen is that it is robust when it comes to overfitting, it is capable of capturing nonlinear relationship and is very good at generalisation. The dataset was subdivided into an 80:20 training to testing split, and the trained models had high predictive accuracy and coefficient of determination (R^2^) of over 0.96 in all the output parameters. Three-dimensional response surfaces were then produced based on the trained digital twin, that depicted the interaction of radiation dose and operational time to determine the effect on V_oc_, J_sc_, FF, and PCE. These surface maps allow continuous performance prediction in the entire operating envelope, which shows how the radiation-induced degradation trends can be predicted with high fidelity by the digital twin.

Besides the data-based systems, the numerical values for each performance metric were also given using formulae based on assumptions advised by specialists. They describe the way output is reduced along a curve, based on dose, time, temperature and other factors such as errors. With the use of simulation, machine learning and mathematics, scientists can create a digital twin that keeps track of ongoing data and forecasts the outcome for solar cells continually facing solar radiation. The approach gives both good predictions and reveals the reason behind failures in components which makes it very useful for terrestrial photovoltaics and satellite power systems.

Besides being data-driven in prediction, every photovoltaic performance metric was directly modelled with physics-informed semi-empirical equations of degradation based on experimental performance and known semiconductor reliability models. The nonlinear or linear relation of the electrical output parameter with cumulative dose of radiation and operating time was developed, occurring secondarily with respect to temperature of operation and a residual error factor that explains stochastic effects. These analytical expressions define the progressive loss processes that are related to the generation of defects by radiation, the decrease in carrier lifetime and the interface degradation.

A hybrid digital twin was developed using physics-based equations of degradation, machine learning prediction, and numerical simulation. This combined method allows to monitor continuously the work of the solar cells and predict in a quantitative manner the behaviour of the solar cells in the conditions of extended exposure to radiations. Notably, the digital twin operates not only as a predictive instrument but also as a mechanistic instrument in the sense that it correlates the manifestations of electrical degradation with the principle underlying the damage mechanisms at the physical level. This ability has found application especially in photovoltaic power systems deployed in terrestrial photovoltaic systems and satellite platforms, where radiation stress over extended durability is important.

In this model, all the photovoltaic parameters were represented as an explicit dependence on radiation dose (G_y_), operating time (h) and temperature (°C): V_oc_, J_sc_, FF, and PCE. The governing equations were built by the combination of the experimentally obtained degradation trends and physically significant coefficients, which made the prediction and interpretation possible. As one example, the open-circuit voltage, or the maximum possible voltage when operating under open-circuit conditions, was modelled to change directly with radiation-induced recombination current and defect density increases, allowing physical damage to be directly related to electrical performance loss.

The V_oc_ was represented by a linear function of the radiation dose D, the length of operation t, and the deviation of the temperature from a reference temperature T_0_ as follows:

1 $${V_{oc}}(D,t,T)={V_{oc0}} - {\alpha _1}D - {\alpha _2}t - {\alpha _3}(T - {T_0})+{\varepsilon _1}$$

where V_oc_ is the initial voltage, α_i_ are degradation coefficients capturing the sensitivity to each variable, and ε_1_accounts for model uncertainty. This form reflects the reduction in quasi-Fermi level separation due to recombination and interface damage under radiation.

Similarly, the short-circuit current density J_sc_ was modelled using:

2 $${J_{sc}}(D,t,T)={J_{sc0}} - {\beta _1}D - {\beta _2}t - {\beta _3}(T - {T_0})+{\varepsilon _2}$$

There are fewer photocarriers produced and collected because radiation produces more recombination centres and decreases the movement of charge carriers in the material.

The fill factor FF, sensitive to series resistance and junction quality, was defined by:

3 $$FF(D,t,T)=F{F_0} - {\gamma _1}D - {\gamma _2}t - {\gamma _3}(T - {T_0})+{\varepsilon _3}$$

where FF_0_ ​is the initial fill factor and the coefficients γ capture losses due to radiation-enhanced contact resistance and interfacial deterioration.

Finally, power conversion efficiency (PCE) was modeled as a compound metric derived from the above three Eq. 

4 $$PCE(D,t,T)=\frac{{{V_{oc}}(D,t,T) \cdot {J_{sc}}(D,t,T) \cdot FF(D,t,T)}}{{100}}$$

With this definition, the model can describe both forms of degradation change when dealing with environmental stresses.

These physics-based degradation models used as performance loss models, as well as testing of the machine-learning predictions. Their combination makes the digital twin framework quite robust and understandable, which is why it will be adequately applied to monitor terrestrial photovoltaic systems long-term. Three-dimensional response surfaces were created to depict how key performance measures are influenced with radiation dose (G_y_) and exposure duration (h) and at constant operating temperature (40 °C).

**Fig. 9:**
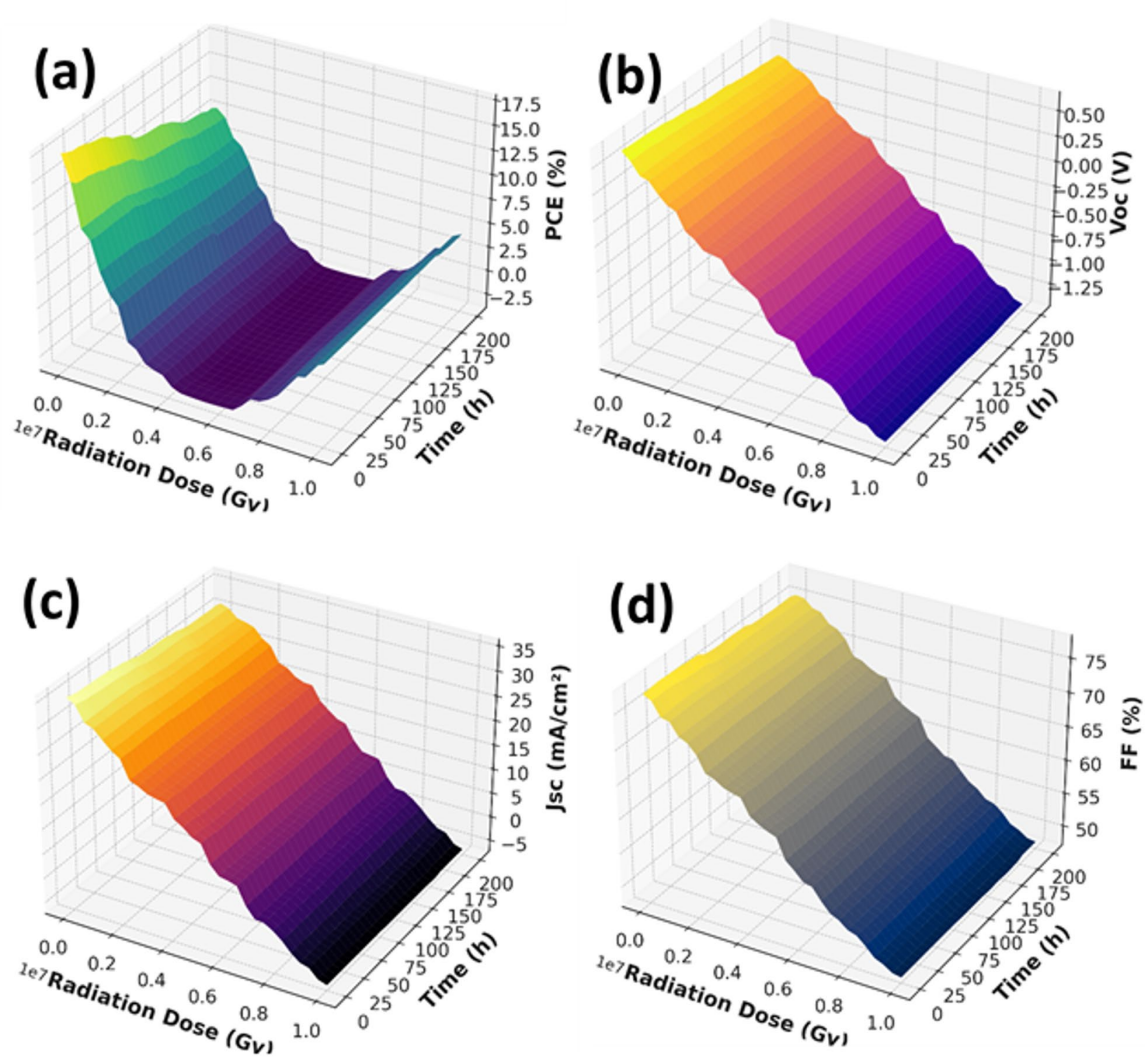
Simulated degradation of graphene-silicon solar cell metrics as functions of radiation dose and exposure time under constant operating temperature of 40 °C: (**a**) power conversion efficiency (PCE) which showed a steep decline with radiation dose and exposure time, (**b**) V_oc_ which was steadily reduced with increased radiation dose and exposure time, (**c**) J_sc_ which declined considerably as a result of a lower charge collection efficiency, and (**d**) (FF) which steadily reduced due to compounded degradation.

As Fig. 9a indicates, PCE decreases nonlinearly and very fast with radiation dose and time of operation. The model forecasts that the PCE values converge to almost zero values in long-term conditions of high-dose exposure, indicating the cumulative values of degraded V_oc_, J_sc_, & FF,. The gradients in the surface plot are steep, which means that the accumulation of radiation-induced damage is nonlinear, especially at higher levels of fluence and at longer exposure times.

Figure 9b is a plot of V_oc_ versus radiation dose and time. V_oc_ steadily drops at a rate that ranges between 0.65 V at the pristine condition to less than 0.50 V in an highest dose and exposure period. This deterioration is explained by the higher recombination losses caused by defects produced by radiation in the bulk of the silicon, and between the graphene and silicon interfaces. The relatively linear relationship between V_oc_ and dose and time validates its appropriateness as the early tracking of radiation damage and imminent equipment failure.

Figure 9c presents the variation of short-circuit J_sc_ reduces significantly from ~ 36 mA cm^−2^ to lower than 10 mA cm^−2^ combined high radiation dose and prolonged operational time. This sharp decrease is caused by the shortening of carrier lifetime, increased recombination and at the interface. This high sensitivity of J_sc_ to radiation and exposure duration makes it an important parameter of diagnosis of radiation-induced performance loss.

The changes in FF are shown in Fig. 9d. FF also has a less steep decrease, dropping to a final value of about 50% at high radiations compared with other performance measurements, that have a starting value of about 78%. This action is in line with radiation-induced series resistance growths, contact degradation, and bi-facial delamination. Although FF does not play a major role in the loss of efficiency at an early stage, the effect of FF increases with the severity of long-term degradation.

On the whole, these findings reveal the potential of the digital twin framework to model photovoltaic performance degradation in terrestrial photovoltaic conditions of high radiation levels using quantitative metrics. The model can predict the change in V_oc_, J_sc_, FF and PCE dynamically by considering the radiation dose and operating time thus offering a useful tool to mission planning, energy control, and early fault identification of photovoltaic systems operating under very severe radiation conditions.

The digital twin model is constructed based on a system of simplifying yet physically inspired assumptions in order to be tractable and interpretable. To remove thermal influences on the performance change in the first case, the operating temperature is kept constant (40 C) to separate thermal influences on performance change in favour of radiation damage; this is reasonable as radiation damage is the leading influence on performance degradation in the conditions studied. Second, radiation exposure is considered uniform over the device area which is understandable due to the controlled irradiation position and homogeneous beam pattern. Third, the assumed degradation mechanisms are cumulative and monotonic with radiation dose and operating time, and ignore the possible self-healing or annealing processes which are negligible at the desired range of temperature. The sensitivity analysis shows that the radiation dose and exposure time are the most significant factors in causing performance degradation whereas changes in temperatures within the range of ± 5 C cause only slight variations in the predicted outputs. It is possible that these assumptions restrict absolute accuracy in highly dynamic thermal or non-uniform radiation fields, but they do not have any effect on the relative degradation profiles or predictive power of the model in the case of long-term radiation exposure scenarios.

To contextualize the performance of Gr-Si solar cell, comparison of the performance of these cells with other available photovoltaic technologies can be useful. Multi-junction III-V and GaAs/InP solar cells have generally high efficiencies (> 28–30%) but still suffer performance degradations of ~ 10–20% at intense levels of proton and electron irradiation in addition to the high material and fabrication costs. Poor ion migration and structural instability limit the improvement of the long-term radiation stability and operational reliability of newly emerging perovskite-based solar cells, although preliminary efficiencies are promising. By contrast, the (Gr-Si) devices explored in this paper maintain silicon-level fabricability, and have much lower degradation compared to this, the Gr–Si devices studied in this paper will retain silicon-level fabricability, with much lower degradation (≈ 2–5%) and thus in practice a trade-off is favourable between efficiency, radiation tolerance and system-level compatibility. Notably, the suggested physics-informed digital twin model has the capability of predictive lifetime evaluation, which is presently deficient in the majority of emergent photovoltaic technologies.

## Conclusion

The paper provides a holistic method of quantifying and predicting the impacts of radiation on graphene-silicon solar cells via physics-based virtual simulation. These heterojunctions can withstand high-energy radiation and that digital twins are best suited to predict photovoltaic performance variations, which is evidenced by the use of experimental know-how, machine learning, and modelling. The graphene and silicon solar cells were far more resistant to protons and electrons and retained nearly 85%of their initial performance following heavy irradiation. Simulated terrestrial photovoltaics up to 10^15^ particles-cm^−2^ performance testing of Graphene-Si cells demonstrated that the cells maintained as much as 85%nof the original PCE, compared to silicon cells which maintained only 55%. The graphene-si devices were also less degraded in V_oc_, Js_c_, and FF at all the radiation fluence levels. With simulated degradation trained on the digital model, it achieved 96.7% of accuracy and gave 3D maps of PCE, V_oc_, J_sc_ and FF degradation in real time. Stress analysis of simulated interface stresses showed that there were hot spots where radiation had destroyed the material, which is in line with the experiment results. Overall, the graphene-silicon solar cells are suitable in terrestrial photovoltaics due to their exceptional radiation resistance. In addition to that, the framework that we established enables us to foresee the fact that the performance will be lowered, provide adequate monitoring, and make decisions in advance in such solar systems.

## Data Availability

The data supporting the findings of this study are available from the corresponding author upon reasonable request.
